# Living through disclosure: a descriptive phenomenological exploration of adult survivors’ experiences of disclosing sexual assault to nurses

**DOI:** 10.1177/17449871261476617

**Published:** 2026-09-18

**Authors:** Claire Dosdale, Mark Bevan, Katy Skarparis

**Affiliations:** Assistant Professor, School of Healthcare and Nursing Science, Northumbria University, Newcastle upon Tyne, UK; Assistant Professor, School of Healthcare and Nursing Science, Northumbria University, Newcastle upon Tyne, UK; Assistant Professor, School of Healthcare and Nursing Science, Northumbria University, Newcastle upon Tyne, UK

**Keywords:** nursing practice, phenomenology, sexual assault disclosure, sexual violence and abuse, trauma-informed care

## Abstract

**Background:**

Sexual assault affects approximately 1 in 3 women and 1 in 18 men in the United Kingdom (UK), yet disclosure to healthcare professionals remains low. Research has focused on prevalence, barriers and outcomes; survivors’ lived experience of the disclosure encounter is under-explored. Nurses’ responses profoundly shape recovery.

**Aim:**

To explore adult survivors’ lived experiences of disclosing sexual assault to nurses in the UK.

**Methods:**

Nine adults who had disclosed sexual assault to a nurse in a UK healthcare setting were recruited and interviewed. Data were analysed using Giorgi’s descriptive phenomenological psychology method.

**Results:**

Disclosure emerged as temporally complex and emotionally overwhelming, shaped by survivors’ profound unpreparedness and pervasive uncertainty. Two themes captured this: (1) Unpreparedness, reflecting lack of readiness before, during and after disclosure and an overriding need for validation and (2) Not Knowing, capturing uncertainty across the practical, relational and emotional dimensions of disclosure. Running through both themes was survivors’ fear of not being believed, which shaped the meaning of the encounter.

**Conclusion:**

Disclosure is a temporally complex, emotionally overwhelming experience fundamentally shaped by nurses’ responses. Belief, validation and agency emerged as central to survivors’ experiences. These findings highlight the importance of trauma-informed education and survivor-centred approaches in nursing practice.

## Introduction

Sexual violence and abuse (SVA) is a deeply violating experience with long-term health implications, cloaked in stigma that frequently inhibits disclosure. SVA encompasses many forms of abuse including rape, sexual assault (SA), child sexual abuse, sexual harassment, forced marriage, honour-based violence, female genital mutilation, trafficking and sexual exploitation and is perpetrated by a stranger (10%) or by someone known and trusted (90%), such as a friend, colleague, family member or partner (Rape Crisis, 2026). This paper focuses on SA as a subtopic of SVA, inclusive of both SA and rape in its use of the term (see Table 1).

**Table 1.** Sexual assault and rape definitions (United Kingdom).

Term	Definition	Key features
Sexual assault	Any intentional sexual touching of another person without their consent, where the touching is sexual in nature (Sexual Offences Act, 2003, Section 3).	Can involve any part of the body or an object.
		Does not require penetration.
		Consent is a key factor.
Rape	Non-consensual penetration of the vagina, anus or mouth with a penis (Sexual Offences Act, 2003, Section 1).	Requires penetration by a penis.
		Lack of consent is essential.
		The perpetrator must not reasonably believe the victim consented.

Sexual Offences Act (2003).

SA affects approximately 1:3 women and 1:18 men in the United Kingdom, yet most experiences go unrecorded, with only 5–34% of survivors disclosing to a formal source (Dosdale et al., 2024). SA does not discriminate by age, sex, religion or class but is a gendered phenomenon, with women significantly more at risk, situating it within a wider context of violence against women and girls. Beyond the individual, SA has ripple effects on partners, children and wider family networks, who often experience secondary trauma, relationship breakdown and caregiving burden as they support survivors through disclosure and recovery (Ahrens and Aldarna., 2012). The response of the recipient is fundamental in reducing health sequelae, including post-traumatic stress (PTS): physical, sexual, emotional and reproductive health problems, alongside social and economic costs (World Health Organisation, 2021). SA-associated PTS is a significant public health concern, encompassing anxiety, social isolation, flashbacks, self-harm and suicide.

Nurses occupy a unique position as frontline professionals who frequently encounter survivors across diverse clinical settings, including emergency departments, general practice, sexual health services and mental health facilities, yet survivors’ experiences are often characterised by negative responses including disbelief, judgemental attitudes and inadequate support (Reeves and Humphreys, 2018).

Despite the critical role nurses play in responding to SA disclosures, significant gaps remain in understanding survivors’ experiences of disclosing to nurses specifically. Nurses’ accessibility, continuity of contact across diverse care settings, and the trust historically placed in the profession (Ipsos, 2025) may position them as a natural recipient of disclosure. Existing research on SA disclosure has focused predominantly on prevalence rates, barriers to disclosure (Wieberneit et al., 2024) and downstream healthcare outcomes (Rajan et al., 2021). Comparatively little is known about survivors lived experiences of the disclosure encounter itself, what it feels like and means, to disclose sexual harassment or assault to a nurse. This study aims to explore survivors lived experiences of disclosing SVA to nurses, using Husserlian descriptive phenomenology to illuminate the essential structures of these experiences. In doing so, the study contributes a deeper understanding of the disclosure encounter that can inform trauma-informed approaches to nursing practice and education.

## Background

### Barriers to disclosure

Multiple barriers impede SA disclosure in healthcare contexts. Survivors report fear of not being believed, concerns about confidentiality, shame and self-blame and uncertainty about how healthcare professionals will respond (Dosdale et al., 2024), compounded by rape myth acceptance and victim-blaming attitudes that persist within society and healthcare systems (Edwards et al., 2011). Many survivors engage in extensive anticipatory thinking before disclosure, weighing potential risks and benefits (Ahrens, 2006).

Internalised blame presents a significant barrier, particularly when experiences do not reflect the societal stereotype of stranger-perpetrated violence. Rape is widely assumed to be violent and anonymous, yet perpetrators are most commonly known to the survivor, who frequently freezes rather than fights back (de la Torre Laso, 2024). The fear of blame as a barrier to disclosing non-stereotypical SA was identified four decades ago and has been consistently replicated since (Ullman et al., 2018; Williams, 1984).

Lack of acknowledgement of the experience as SA presents a further barrier. Koss et al. (1988) found that 70% of women who described unwanted sex did not identify it as rape (‘hidden rape’), and survivors are more likely to seek formal support when their experience aligns with stereotypical SA, including the presence of physical injury (Ahrens et al., 2010).

Men are consistently less likely to disclose SA, with barriers rooted in pervasive rape myths that: men cannot be raped, real men can defend themselves and male rape only occurs in prisons (Turchik and Edwards, 2012). Shame, guilt and the perceived female-orientation of support services compound these barriers (Allen et al., 2015), and male SA remains a largely hidden crime (Walfield, 2018).

Diversity adds further complexity. Minoritised women are less likely to report SA than white women, with distrust of public officials a persistent barrier (Hakimi et al., 2018), whereas cultural factors including family honour and shame create additional barriers globally (Huong, 2012). Older adults represent another underserved group, their experiences rendered invisible by data collection practices that reinforce assumptions of asexuality (Bows and Westmarland, 2016).

The literature demonstrates that disclosure barriers are deeply intersectional, highlighting an urgent need for nursing practice that addresses these overlapping systems of oppression.

### Healthcare responses to survivor disclosure

When survivors disclose, healthcare professionals’ (HCP) responses profoundly impact their experiences and ongoing engagement with services: positive responses characterised by belief, empathy and non-judgemental support facilitate healing, whereas disbelief, blame or dismissiveness can re-traumatise survivors and deter future disclosure (Dosdale et al., 2024).

Research consistently identifies that HCPs lack sufficient knowledge and confidence to respond effectively to SA disclosures. Vandenberghe et al. (2018) found that whilst over half of healthcare staff felt comfortable exploring SA in consultations, many reported insufficient knowledge of its prevalence and noted their own wellbeing could affect care quality.

Rape myth adherence among HCPs presents a significant barrier. Ranjbar and Speers (2013) found that survivors overwhelmingly reported negative healthcare encounters, citing inexperience, disrespectful treatment and adherence feeling ashamed to SA stereotypes. Rape myth-driven blame was the most consistent barrier, with minimising and dismissive responses further compounding negative experiences (McQueen et al., 2021).

Mental health settings present particular concerns. McLindon and Harms (2011) found that over 95% of mental health nurses believed patients should not be asked about sexual trauma, and 73% had received no relevant training in over 10 years. Given that approximately 40% of women attending sexual assault referral centres are known to mental health services, this reluctance is especially troubling (Hughes et al., 2019).

Positive disclosure experiences are consistently associated with validation, empathy and being believed (McQueen et al., 2021). Henninger et al. (2019) demonstrated that being treated with respect and cultural sensitivity mattered more to survivors than procedural clarity alone.

### Gaps in current knowledge

Healthcare settings represent important disclosure contexts, as they are often one of the few space’s survivors access repeatedly and regardless of readiness to disclose formally elsewhere, offer confidential and professionally facilitated opportunities for disclosure that may not exist in other areas of survivors’ lives. Yet survivors frequently report unsatisfactory experiences with healthcare professionals. Understanding survivors’ experiences of disclosing to nurses is essential to encouraging disclosure, improving responses and aiding recovery, enabling nurses to signpost survivors to timely, appropriate help and reducing the psychological and physical impact of SA. This study addresses this gap, with findings intended to inform practice, enhance survivor outcomes and contribute to a trauma-informed evidence base for nursing education and clinical care.

Research question:

- What is the experience of survivors of SA in making disclosures regarding their assault to nurses?

The intended aims are to:

- Develop an insight into survivors’ SA disclosures in a healthcare setting.
- To offer an original research contribution in understanding experiences SA disclosures in nursing practice.

## Methods

### Design

This study employed Husserlian descriptive phenomenology, specifically Giorgi’s (2012) Descriptive Psychological Phenomenological Method. This perspective, rooted in the work of Husserl (1970), asserts that reality is not fixed but is experienced subjectively by individuals within their social environment, concerned with lived experience and how consciousness engages with the world in an embodied and situated way.

The study used a non-dyadic design: survivor and nurse participants were recruited independently and did not share the same disclosure encounter. This paper focuses on the survivor experiences. The adherence to consolidated criteria for reporting qualitative (COREQ) guidelines has been followed (Tong et al., 2007; see Supplemental Material for checklist).

The phenomenological reduction (epoché), the deliberate suspension of judgement in which the researcher’s taken-for-granted assumptions, beliefs and prior knowledge are set aside or ‘bracketed’, was employed throughout data collection and analysis to bracket the researcher’s assumptions and pre-existing knowledge, allowing phenomena to be explored as they present themselves to consciousness (Husserl, 1970).

### Study setting and recruitment

Purposive sampling recruited survivors of SA (*n* = 9) through a regional higher education institution. Of those, eight identified as female and one as male. All had self-defined experiences of SA and had disclosed that experience to a nurse, meeting the inclusion and exclusion criteria detailed in Table 2. Sample size was guided by the phenomenological concept of sufficiency rather than a predetermined number, with the aim of capturing rich, in-depth description of the phenomenon across a range of participant experiences. In line with Giorgi’s (2012) approach, recruitment continued until the essential structures of the experience had been described with sufficient depth and clarity. This resulted in a final sample of nine participants, whose accounts offered detailed descriptions into the phenomenon under study. This reflects the broader phenomenological priority of depth over breadth, where the quality and richness of experiential description take precedence over sample size or statistical generalisability.

**Table 2.** Participant inclusion and exclusion criteria.

Inclusion criteria	Exclusion criteria
Self-defined experience of SA	The participant is under 18 years old (*This study focuses on adult experiences of disclosure.*)
18 Years old or over at the age of experience and at participation.	The SA took place when the participant was under 18 years old. (*Safeguarding implication and due to the adult focus of the study.*)
Able to have the mental capacity to consent to participate in the research study	There are current legal proceedings regarding the SA discussed (*so as not to potentially jeopardise ongoing legal cases*).
Disclosed their episode of SA to a nurse	Where the perpetrator of the SA discussed is identified as a current partner (*for all parties’ safety*).

SA: sexual assault.

Survivor participants ranged in age from 19 to 51; eight identified as female and one as male.

### Data collection

In-depth phenomenological interviews were conducted using Bevan’s (2014) method, which structures the interview across three phases: contextualisation (eliciting the lifeworld in natural attitude); apprehending the phenomenon (focusing on the specific disclosure experience) and clarifying through imaginative variation (exploring different dimensions and aspects of the experience to identify what is essential to its structure). This structure facilitated rich description while maintaining the phenomenological attitude throughout, ensuring that the researcher’s follow-up questions clarified participants’ meanings rather than introducing the researcher’s own assumptions. An example can be found in Table 3.

**Table 3.** Bevans (2014) structure of descriptive phenomenological interviewing.

Phenomenological attitude	Research approach	Interview structure	Method	Example question
Phenomenological reduction (epoché)	Acceptance of natural attitude of participants	Contextualisation (eliciting the lifeworld in natural attitude)	Descriptive/Narrative context questions	‘tell me about how you came to disclosure your experience to a nurse’, ‘Tell me what made you feel able to disclose’
	Reflexive critical dialogue with self	Apprehending the phenomenon (modes of appearing in natural attitude)	Descriptive and structural questions of modes of appearing	‘tell me about your journey to the clinic that day’, ‘tell me about your time in the waiting room waiting to be called’
	Active listening	Clarifying the phenomenon (meaning through imaginative variation)	Imaginative variation: of structure questions	‘You mentioned you felt judged in the waiting room, can you tell me a little more about what you mean by ‘judged’, ‘You talked about the nurse confirming you had been assaulted, can you tell me a little more about that conversation’

Interviews were audio-recorded, lasted between 60 and 150 minutes and were conducted in private settings chosen by participants. Transcripts were produced verbatim for analysis and all participants were given pseudonyms for confidentiality.

### Data analysis

Analysis followed Giorgi’s (2012) five-step descriptive phenomenological psychological method. Transcripts were read multiple times to achieve holistic understanding before analytical reduction began. Transcripts were then re-read with attention to transitions in psychological meaning, marking natural breaks where significance shifted; this was undertaken by hand to maintain immersion in the data. Each meaning unit was transformed from participants’ natural language into descriptive psychological language, maintaining fidelity to their descriptions while revealing psychological significance, with free imaginative variation applied at this stage. Meaning units were subsequently synthesised into general structural descriptions, revealing patterns across participants, before final synthesis into themes representing the essential constituents of experience for each participant group independently.

**Figure 1.**
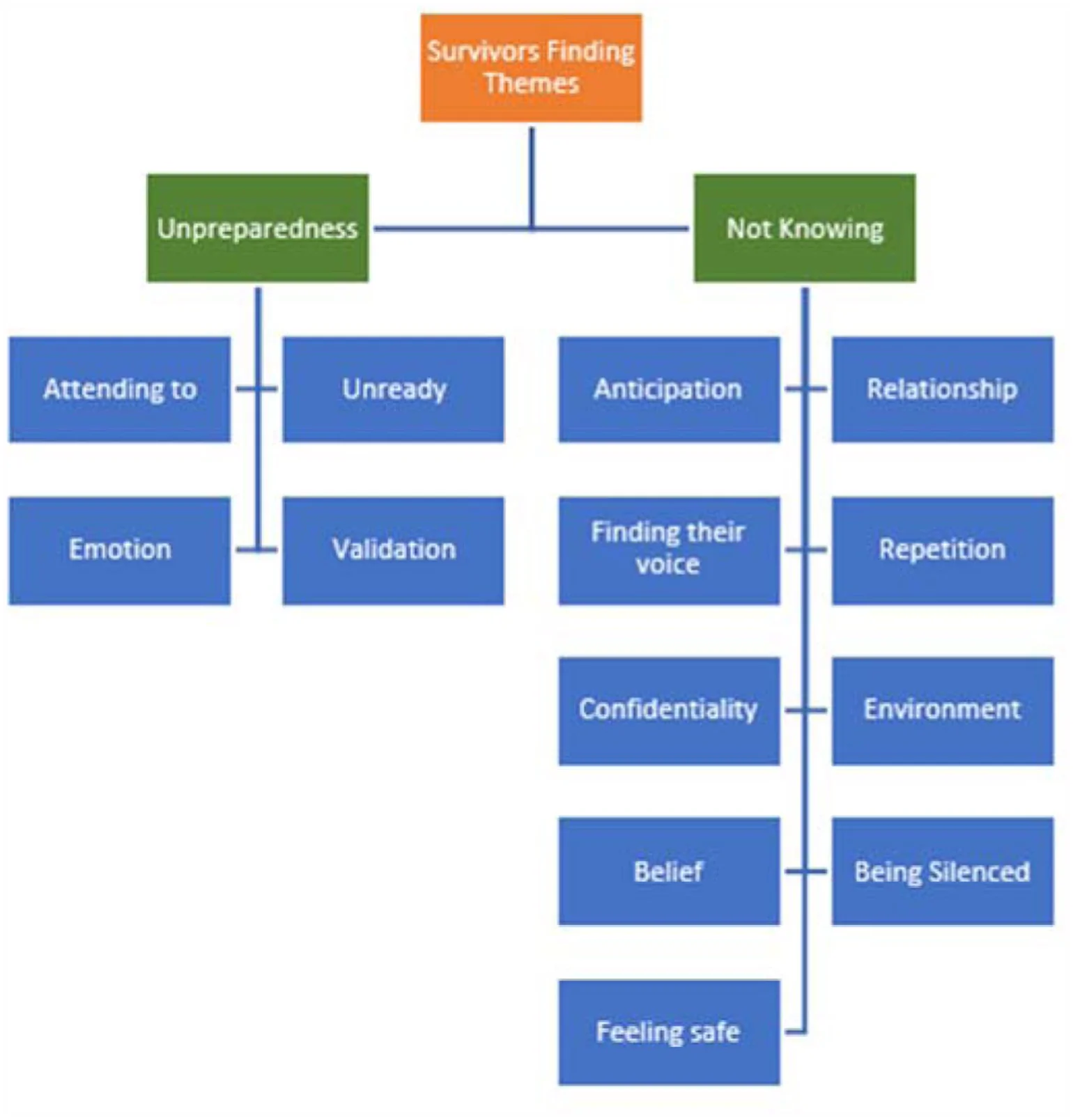
Survivor finding themes.

This process generated 68 essential structures across survivor participants’ accounts. Following Giorgi’s (2012) descriptive phenomenological psychological method, these structures were progressively reduced through iterative comparison and clustering of units sharing a common psychological meaning, with free imaginative variation applied to distinguish essential from incidental features of the experience. This synthesis produced 13 sub-themes, and ultimately two overarching themes representing the essential constituents of the disclosure experience (see Figure 1).

### Ethical considerations

Ethical approval was granted from Northumbria University (reference number HLS-PHW151611). All participants provided written informed consent. The study was undertaken 2016–2022 with data collection 2019–2021.

All participants provided written informed consent prior to participation. Given the sensitive nature of the topic, a detailed risk management protocol was developed, including: (1) provision of a debriefing sheet with support resources; (2) regular check-ins during interviews to monitor participant wellbeing; (3) the option to pause or withdraw at any time without consequence; and (4) referral pathways to specialist support services if required. Data were anonymised using pseudonyms, stored on a secure university server accessible only to the research team and retained in accordance with institutional data management policies. The decision not to use member checking was made to minimise risk of re-traumatisation for survivor participants, consistent with Giorgi’s (2012) analytical method, which does not require participant validation of derived structures.

### Rigour, trustworthiness and reflexivity

Trustworthiness was established using Lincoln and Guba’s (1985) framework, without member checking. Credibility was supported through prolonged engagement with the data, transparent sampling, adherence to Giorgi’s method and reflexive journaling. Confirmability was addressed via a clear audit trail of methodological decisions, raw data preservation and regular debriefing with an experienced descriptive phenomenological researcher. Dependability was ensured through detailed documentation of all analytical steps. Transferability is facilitated by rich, thick description and extensive participant quotation, enabling readers to assess applicability to their own contexts. Participant and setting demographics are reported in the findings to support contextual judgement by other researchers.

The lead researcher’s clinical background in sexual health nursing presents both strengths and potential limitations. This expertise facilitated rapport and understanding of clinical contexts but may have introduced assumptions about the phenomenon. To mitigate this, phenomenological reduction (epoché) was employed throughout, and regular debriefing with a supervisor experienced in descriptive phenomenology was conducted to examine potential biases. The researcher maintained a reflexive journal documenting assumptions, emotional responses and analytical decisions to enhance transparency

## Findings

Two themes emerged from survivor participant data: (1) Unpreparedness and (2) Not Knowing, capturing the temporal complexity of disclosure as it extends before, during and after the disclosure moment rather than being confined to the act of telling. These themes are closely interrelated: Unpreparedness reflects survivors’ internal, embodied orientation, their own readiness to confront and process the experience, whereas Not Knowing reflects the situational and relational uncertainty of the disclosure encounter itself, particularly in relation to the unknown nurse, environment and procedures. Rather than occurring in sequence, the two themes compound one another throughout the disclosure timeline: profound unpreparedness heightens the threat posed by situational uncertainty, whereas pervasive not knowing continually undermines survivors’ capacity to feel ready, together shaping disclosure as a sustained experience of vulnerability.

### Theme one: Unpreparedness

Unpreparedness captures survivors’ profound lack of readiness for what follows the experience of SA. Participants described feeling unprepared at every stage: before, during and after disclosure. This theme encompasses four sub-themes: attending to, unready, emotion and validation.

#### Attending to

Survivors described the internal struggle between hiding from their assault experience and confronting it, initially turning away using coping mechanisms that served a self-care function but created internal conflict. Jane articulated this struggle: ‘*I sort of knew that what had happened wasn’t quite right [the assault], but I’d sort of shrugged it off and never really done anything about it*’ (Jane). This quote illustrates the tension between knowing something was wrong and being unable or unwilling to fully acknowledge it.

This is typified by Vicky as she explored the way the rape impacted her behaviour and how unprepared she was for it to do so. The experience caused more emotion than she had been prepared for, and she attributed this to her change in behaviour. She was able to recognise this behaviour was a consequence of the rape. Subsequently, recognising it prepared her to disclose in order to seek support: ‘*I think I’d been more stressed and worried about it than I thought, and I think it was affecting my behaviour because I didn’t feel that it had been acknowledged in any way and it was quite serious and . . . So I’d begun drinking and I think it was actually kind of through that that I decided to erm . . . think about telling*’ (Vicky). The decision to *attend to* their experience, to face it rather than hide from it, represented a significant psychological shift. However, this *attending to* did not equate to feeling prepared. Instead, it marked the beginning of confronting profound unpreparedness for what disclosure would entail.

#### Unready

Participants emphasised feeling unready to navigate the disclosure process. This unreadiness manifested in multiple ways: not knowing what to expect, lacking preparation for the emotional intensity of the disclosure and being unprepared for the responses they might receive. Lucy described this: ‘*Like I’m very, very, very guarded and I just thought like: I’m worried in case I end up crying or something like this, like in front of a stranger, ’cos I really just feel really . . . Like I would be feeling too exposed*’ (Lucy).

The vulnerability inherent in disclosure, combined with lack of preparation, created significant anxiety. The unreadiness extended beyond the disclosure moment itself. Survivors felt unprepared for what might follow, police involvement, forensic examinations and ongoing healthcare interactions. Sarah expressed this: ‘*I didn’t know what was going to happen. I just didn’t know*’ (Sarah). This lack of information and preparation compounded the difficulty of disclosure.

It was also noted that post-disclosure experience ended up becoming a part of the SA experience: ‘*you’re reliving that all the time and then it ends up getting just merged into one little Groundhog Day of what’s happened during your rape and what’s happened afterwards*’ (Sarah). This structure of experience is explored further within the not knowing theme; experience is interlinked and does not break into neat subthemes.

#### Emotion

The emotional overwhelm associated with disclosure represented a central aspect of unpreparedness. Participants described intense, complex emotions that they felt ill-equipped to manage. These emotions included fear, anxiety, shame, guilt, anger and sadness, often experienced simultaneously. Sally powerfully captured this emotional intensity: ‘*Angry. Like nobody believed me. Erm, I didn’t know what to do. Erm, I didn’t want to walk out of there and then possibly do something, like harmful or anything like that, erm, so . . . I was like: no. I need to tell somebody. Somebody needs to help me before I get to that point*’ (Sally).

For some, the emotional response to disclosure extended beyond immediate feelings. Anne described emotional numbing: ‘*But it wasn’t erm . . . I had a reaction to it where I just completely switched off, but I didn’t really get any . . . I didn’t really get that much of an emotion. Then I just felt . . . emotionally, I felt nothing*’ (Anne). This emotional detachment not only served as a protective mechanism but also illustrated the profound psychological impact of disclosure.

Conversely, when met with dismissive or disbelieving responses, emotions intensified. ‘*And I completely felt like: that’s just where I completely felt like, ashamed of whatever. And you know, it’s a normal feeling to coincide with that – that heightened . . . Me feeling ashamed of that situation, I think I was already embarrassed by it, but then it completely shut anything down for me disclosing it to anyone else*’ (Holly).

These emotions can manifest in physical reactions, reflecting embodiment – a whole-body lived experience rather than a mind-body split. Three participants described emotional overwhelm manifesting visibly in the lead-up to disclosure: ‘*I just remember feeling sick*’. (Jane); ‘*I felt sick. I just felt really sick*’. (Sarah); ‘*Just felt sick the entire time*’. (Holly). All three describe ‘feeling sick’, displacing emotional overwhelm into a bodily response – making the impact of their experience physically unquestionable, even when the experience itself felt questionable to them.

#### Validation

Validation captures survivors’ own difficulty in acknowledging and processing their experience without external confirmation, reflecting a further dimension of their unpreparedness. Many survivors described feeling unable to fully accept or make sense of what had happened to them until someone else confirmed it; this need for external validation illustrates how unprepared survivors were to process the experience themself. Jane described seeking validation from someone with professional knowledge:

‘*Yeah, it was like immediate. It was like ‘yes. That is a sexual assault. You did not have the capacity to consent’ and it was quite like … Straight. Which I think is what I needed to hear. that’s when I like, fully broke down and was like crying and … I think … It was kind of like my moment of realisation that I knew that she was going to say that that was what it was and … Yeah. It was instant. And I kind of said to her, like … You know … ‘It kind of wasn’t really real ‘til you said that*’ (Jane).

When validation was received, it facilitated survivors’ ability to acknowledge their experiences and move towards healing: ‘*. . . Not that I didn’t want to tell her [the nurse] . . . I just . . . I hadn’t really processed what had happened myself to even tell her that I didn’t really understand what had happened, so for me, it was like . . . a big realisation when she confirmed it. I needed that*’ (Holly).

However, lack of validation had profound negative consequences. Anne described how a nurse’s questioning led her to doubt her own experience: ‘*It’s like a ball that you can’t stop rolling once you’ve even mentioned something. But then it made me think, like oh, maybe I shouldn’t have come . . . she [the nurse] said some things and it made me like really question it [the experience]*’ (Anne). This quote illustrates how lack of validation can lead survivors to question the validity of their own experiences, compounding trauma.

### Theme two: Not knowing

Not Knowing captures the pervasive uncertainty that characterised survivors’ disclosure experiences. This theme encompasses four sub-themes: belief; the emotional labour of disclosure (anticipation, finding their voice and repetition); the relational dynamics of disclosure (relationship and feeling safe) and loss of agency and control (choice, being silenced, confidentiality and environment).

#### Belief

Running through each of these sub-themes was survivors’ fear of not being believed, the most dominant concern shaping the entire disclosure experience fear of not being believed emerged as the most dominant concern across participants’ experiences, pervading all aspects of disclosure. Survivors described extensive anticipatory worry about whether nurses would believe them, scrutinising nurses’ reactions for signs of belief or doubt Jane expressed this fear: ‘*I was worried that I was going to go and even though I knew that it was real, that someone might say ‘well actually, no. It wasn’t’’*. This fear existed even when survivors themselves knew their experiences were real, highlighting the power dynamics inherent in disclosure. When belief was communicated, through words, body language or actions, survivors experienced profound relief. However, when disbelief was perceived, even subtly, the impact was devastating and often deterred future disclosure.

#### The emotional labour of disclosure

Disclosure demanded considerable emotional effort from survivors, encompassing anticipatory anxiety, the struggle to find words for the experience and the burden of repeating their account multiple times.

Anticipation suffused the entire disclosure experience. Participants described intense anticipatory anxiety about how nurses would respond, what questions would be asked, what procedures might follow and who might find out. Lucy articulated this: ‘*Again, just like on edge, you know? Just sort of like: aah, what’s going to come out here? Am I going to cry, or like what’s going to happen here*’ (Lucy).

The future-oriented nature of anxiety meant survivors were constantly projecting into unknown futures, imagining worst-case scenarios. ‘*I was worried that I was going to go and even though I knew that it was real, that someone might say ‘well actually, no. It wasn’t’ and be like ‘this didn’t happen to you, so why are you here crying about it?’ Sort of thing*’ (Jane). This anticipatory anxiety also contributed to the embodied experience: ‘*Getting on the metro with the other half and it was just . . . The longest metro journey ever, just thinking. Sitting in the waiting room . . . Walked around from the metro station It was just really, really upsetting. Really slow walking across the road*’ (Sarah). Time seemed to distort under the weight of anticipatory anxiety, with moments stretching unbearably.

Many participants described difficulty in knowing how to articulate their experiences, finding the words to describe what had happened. The assault represented an ‘alien’ experience for which ordinary language seemed inadequate. Sally captured this: ‘*I didn’t speak for like half an hour, ’cos I just, like, physically find it hard to say things out loud*’ (Sally). The physicality of this difficulty, not just psychological but embodied, illustrates the profound challenge of voicing assault experiences. Jane described similar difficulty: ‘*I was really anxious and really like . . . Planning what I was going to say and how I was going to say it and like, you know when it’s going like over and over and over in your head?*’ (Jane).

Fear of disbelief compounded the difficulty of finding words, creating a paralysing cycle for re-living the experience. Survivors experienced disclosure as repetitive and re-traumatising, not knowing this was going to be an aspect of their disclosure meant they were unaware they would be re-living their experiences multiple times to different healthcare professionals. One participant (*Sarah*) described having to disclose ‘*to 3 people in one visit*’, which was described as ‘*emotionally difficult*’. This repetition served institutional and procedural needs rather than survivor needs, highlighting a system-centred rather than survivor-centred approach. ‘*Well each time, I just . . . I felt more ashamed and disappointed with myself*’ (Jack). The planning and rehearsing required for disclosure also contributed to its traumatic repetition. One participant described:

‘*Because you’d had to relay it so many times. Like, I had to tell a police officer like, in general, just . . . and then I had to tell them again, so they could write down exactly what I’d said and then I had to go out to the place and tell the nurse and because I’d relayed it so many times, it was just coming off naturally, but after you get questioned . . . It was a little bit intimidating, ’cos she was like asking questions that like, implied that they were going to trick you*’ (Anne).

This internal repetition added to the burden of disclosure.

#### The relational dynamics of disclosure

Not knowing the nurse to whom they would disclose shaped survivors’ experiences profoundly, as they searched for signs of safety and connection in an unfamiliar relationship. Not knowing the nurse to whom they would disclose significantly amplified anxiety; survivors scanned for signs of kindness, empathy and safety, with no established relationship from which to predict how disclosure would be received. Jack expressed concern about encountering someone known to family: ‘*I mean I could hear her walking along the corridor, so . . . ’Cos you don’t know who it is and again, my mam being a nurse, I don’t know if she had any friends working in that area and if she had, it would have been awful, embarrassing, I felt sick*’ (Jack). This quote highlights how not knowing who they would encounter created multiple layers of anxiety. ‘*All of the staff were dressed in their own clothes. There was no distinguishing who was a doctor, who was a nurse, who was a healthcare and it made me feel confused*’ (Sarah).

Survivors sought maternal or caring qualities in nurses, associating these with safety and belief. ‘*Erm, came and got us and I just remember thinking: oh, she looks really friendly. She had like a nice smile; she had really nice ginger curly hair; I remember that*’ (Jane). This search for recognisable caring attributes reflects survivors’ need for predictability in an unpredictable situation. Survivors described seeking and needing to feel safe during disclosure, in a situation where not knowing what was coming next seeking safety, or what the perceived as safety, was a core aspect of their experience. Feeling safe was closely linked to being believed and not being judged. Participants associated safety with particular nurse qualities kindness, empathy, calmness and a non-judgemental demeanour. ‘*But she was very like . . . Erm . . . Caring, the whole time. Very, erm, patient. I felt quite reassured by it*’ (Jack). A maternal quality was clearly valued; Vicky and Jack typified this: ‘*She was kind of like ‘motherly’ really, in a sense*’ (Vicky); ‘*. . . and I think she was very [comforting] and accepting and quite motherly and she was nice*’ (Jack). Survivors expressed that a compassionate response made them feel safe, believed and supported. Sally and Vicky characterised this: ‘*There was something that meant I felt safe enough to talk to her and I’m not sure . . . Erm . . . Kind of almost like . . . I think it felt like she was on my side . . . I haven’t often kind of felt that able to speak to somebody or also looked after in the . . . You know, within what happened. So almost it felt like she was going to help me get some help*’ (Vicky); ‘*She was just very, very, very like, sympathetic, like just very nice*’ (Sally).

#### Loss of agency and control

Survivors described a pervasive lack of control over their disclosure experience, from the physical environment and confidentiality of the encounter to their ability to be heard or decline aspects of care.

Lack of choice following disclosure emerged as a significant concern. Survivors described feeling they had little control over their future was attributed to the specifics, or practical elements, of the consultation: ‘*Cos she’d told me before I’d even gone into the room and then once you get into the room, erm, a police officer who comes down like . . . Round the curtain . . . Erm, so I had to agree*’ (Anne). ‘*I just didn’t want to get undressed. I didn’t want to expose myself to strangers again. And I just really wasn’t prepared for it and I didn’t know whether or not I could refuse. Yeah, I didn’t know whether I could refuse to disclose to the nurse and just say ‘look, I’m not very comfortable; I’ve gone through a . . . I thought this was part of the process*’ (Sarah). There was a lack of awareness that any part of their consultation could be declined, and an acceptance and trust that these elements were just what was to happen.

Some survivors experienced being silenced through dismissive responses, questions that suggested blame, or healthcare professionals cutting short their accounts, often not knowing they could pause or refuse aspects of care. Holly described a dismissive response: ‘. . . so she said that in these circumstances, there would be no point in going to the police; it would be my word against his and it would probably cause more harm than good’ (Holly). Being silenced not only affected the immediate interaction but had lasting consequences for help-seeking. ‘No. I just wanted to get out of there. I didn’t want to see anyone. I thought In my head, I thought they all know what’s happened now. They’re judging me and I just wanted to get out’ (Sally). Participants feared this response and often their fears were validated, which led to disappointment because, although they had prepared for the response, they had not hoped for it. Jack described his experience and used the word ‘victimised’ to further describe how he felt about the nurse’s response: ‘victimised in a way and . . . it’s all wrong, As if they’d heard it before’ (Jack). Judgemental or dismissive responses solidified survivors’ existing fears of being blamed, heightening emotion and frustration. This shaped the remainder of the consultation, with a lack of trust creating communication barriers and contributing to survivors wanting to leave, ultimately being silenced.

Concerns about confidentiality pervaded survivors’ experiences. These concerns operated on multiple levels: worries about who else in the healthcare system might find out, concerns about information sharing with police or other agencies and fears about the impact on the perpetrator. ‘*But he had children as well and if something happened, then obviously it’s something that would be taken very seriously. And obviously something will come of it, whether it goes to court or it doesn’t; something will come of it and then I didn’t want people to find out*’ (Anne). Another participant also touched on this from a wider perspective; demonstrating that in a time of extreme emotional pain, survivors consider the impact of their assault on other people. This adds another level of responsibility to their anxieties: ‘*And it would ruin many other people’s lives other than just mine*’ (Jane).

Participants described acute awareness of their environment, who might overhear, who might see documentation, whether conversations were truly private. This hypervigilance added to the stress of disclosure. ‘*The colours [in the waiting room] weren’t very welcoming; but it was more the fact that there was other people there*’ (Jack). The physical environment significantly impacted disclosure experiences. Participants described heightened sensory awareness noticing corridor layouts, waiting room configurations, the presence of other people and the physical space of consultation rooms. Sarah’s description illustrates this embodied environmental awareness: ‘*A whole melting pot of emotions and you just try and keep it in check, because there’s other people around you and it’s just not a very nice environment to be in, because I think . . . Because I didn’t know what was going to happen. I just didn’t know*’ (Sarah).

The psychological weight of disclosure altered as they spent more time in the clinical area: ‘*It was busy and it was loud. Well I thought it was louder. Like, everything was just amplified. Erm . . . And then my name beeped and I thought: can I bottle out of this? Can I just go? But then in my head, I cannot stand people wasting appointments, so I was like: no, I need to do this, so I went in*’ (Sally). Environments that felt exposed, busy or clinical created additional barriers. However, survivors often had no control over the environments in which they found themselves disclosing.

While the themes of unpreparedness and uncertainty characterised the disclosure experience for all participants, some variation was evident in how this unfolded. Rather than a positive experience from the outset, participants such as Vicky, Jack and Sally described a shift within the encounter itself, once the nurse demonstrated caring, non-judgemental qualities, feelings of safety and being believed began to emerge, even where anticipatory anxiety and uncertainty had dominated up to that point. This suggests that while unpreparedness and not knowing may be near-universal at the outset of disclosure, supportive nurse responses can shift the trajectory of the encounter as it progresses, underscoring the importance of individualised, trauma-informed care at every stage of the interaction, not only in preparation for disclosure

## Discussion

This study provides an in-depth phenomenological exploration of adult survivors’ experiences of disclosing SA to nurses in healthcare settings, revealing disclosure as profoundly complex and emotionally overwhelming, characterised by unpreparedness and pervasive uncertainty. The relationship between the two themes reflects the dual structure of disclosure as both an internal and a relational experience. Unpreparedness captures survivors’ own embodied orientation towards the experience, their internal readiness or lack of it to confront and process what had happened. Not Knowing, by contrast, reflects the situational and intersubjective uncertainty of the encounter itself, most acutely the unknown nurse, environment and procedures survivors faced. These structures were not sequential but mutually reinforcing survivors’ lack of internal readiness intensified the threat posed by external uncertainty, whereas that uncertainty, in turn, prevented survivors from ever fully closing the gap in their own preparedness. Together, they characterise disclosure as a sustained, compounding experience of vulnerability, rather than a single moment of telling.

### The centrality of belief

The most prominent finding is survivors’ overwhelming fear of not being believed. This finding aligns with broader literature identifying belief as a critical factor in survivors’ post-assault adjustment (Ullman and Peter-Hagene, 2014), while extending it by revealing the phenomenological depth of belief, not merely as a cognitive judgement, but as an intersubjective experience that fundamentally shapes the meaning of disclosure itself. Where previous work has largely measured belief as an outcome variable linked to psychological adjustment (Ullman and Peter-Hagene, 2014) or examined its absence in the context of police responses (McQueen et al., 2021), the present study extends this by showing how the fear of disbelief operates continuously throughout the disclosure encounter, rather than as a single evaluative moment. This difference may reflect the phenomenological approach taken here, which captures the lived, moment-to-moment texture of anticipating and interpreting belief, something less visible in outcome-focused or quantitative designs.

From a phenomenological perspective, belief validates the survivor’s lived experience: when nurses communicate belief they affirm survivors’ reality, facilitating acknowledgement and processing. Conversely, disbelief invalidates experience, leading survivors to question whether assault had occurred and deterring future engagement with support services.

### Unpreparedness and trauma

Survivors’ profound unpreparedness reflects both individual and systemic factors. At an individual level, SA represents a rupture in the lifeworld, creating an ‘alien world’ (Steinbock, 1995). At a systemic level, unpreparedness reflects inadequate information about what to expect when disclosing and lack of clear communication about disclosure processes, creating unnecessary anxiety and barriers to care. Much of the existing literature on disclosure has focused on why survivors delay or avoid disclosure altogether (Ahrens, 2006; Dosdale et al., 2024), rather than on survivors lived experience of unreadiness once they have made the decision to disclose. This study extends that literature by showing that unpreparedness does not resolve once disclosure begins; rather, it persists and evolves across the encounter, from the decision to attend to the experience, through to processing its aftermath. This may reflect a genuine difference in focus rather than a contradiction: prevalence and barriers research typically captures the pre-disclosure decision point, whereas this study’s phenomenological lens captures unpreparedness as a continuous, unfolding structure of experience.

The emotional overwhelm described by participants reflects the re-traumatising potential of disclosure; when survivors recount their experiences, they relive the trauma. The repetitive nature of disclosure compounds this, supporting growing recognition of the need for trauma-informed approaches that minimise repetitive questioning and prioritise survivor wellbeing over procedural convenience (Quadara, 2015).

### The intersubjective nature of disclosure

Disclosure emerged as fundamentally intersubjective, shaped by the relationship between survivor and nurse. Survivors searched for signs of empathy, interpreted nurses’ responses and adjusted their own expressions accordingly. Previous research has tended to examine nurses’ and other professionals’ perspectives on responding to disclosure (McLindon and Harms, 2011) or survivors’ broader satisfaction with services (Henninger et al., 2019), rather than the moment-to-moment relational dynamic from the survivor’s own perspective. The present findings suggest this dynamic is more fine-grained than previously captured, survivors were shown to interpret subtle, embodied cues, tone, facial expression and demeanour, in real time, and adjusted their own disclosure accordingly. This difference likely reflects methodology: professional-perspective or satisfaction-based studies are less able to capture the survivor’s continuous, embodied interpretation of the nurse as it unfolds within the encounter itself.

This highlights the importance of nurses’ communication skills and self-awareness; facial expressions, body language, tone and word choices are all perceived by survivors, and even subtle signs of discomfort, disbelief and the environment can profoundly impact the disclosure interaction.

### Lack of agency and choice

The finding that survivors experienced limited agency contrasts sharply with trauma-informed principles emphasising the rebuilding of control (Hopper et al., 2010). Many participants described feeling swept along by institutional procedures, with this lack of agency mirroring the loss of control experienced during assault and potentially re-traumatising survivors. This contrasts with trauma-informed care literature, which largely frames the restoration of choice as an achievable organisational goal (Hopper et al., 2010); our findings suggest that in practice, procedural and systemic demands can override these principles even where trauma-informed intentions exist. This gap between principle and lived experience may reflect the fact that much existing trauma-informed care literature is conceptual or policy-focused, whereas this study captures survivors’ first-hand experience of care as it was actually delivered.

## Strengths and limitations

This study offers several notable strengths. It provides an in-depth phenomenological exploration of survivors lived experience of disclosing SA specifically to nurses, addressing a significant gap in a literature that has predominantly focused on prevalence, barriers and outcomes. A key strength of this study lies in the depth of insight it provides into survivors’ own lived experience of disclosure, an experience that has been largely absent from a literature dominated by prevalence rates, disclosure barriers and downstream healthcare outcomes. By centring survivors’ own accounts, this study reveals the temporal complexity of disclosure as it extends before, during and after the moment of telling, rather than treating disclosure as a single, discrete event. It illuminates the profound unpreparedness and uncertainty survivors experience throughout this process, and the centrality of belief, validation and agency to how disclosure is felt and understood from the survivor’s own perspective. This offers a level of experiential depth not accessible through quantitative or outcome-focused designs, providing insight into what disclosure actually feels like and means for survivors themselves, insight that can meaningfully inform how nurses understand and respond to disclosure in practice.

The use of Husserlian descriptive phenomenology, a method comparatively underused in this field relative to quantitative and outcome-focused designs, demonstrates the additional depth this approach can offer in understanding survivors’ experience.

Participation was voluntary, and it is likely that those who came forward were survivors who felt more ready or able to talk about their experiences; some survivors may have self-selected out of the study, meaning the range of experiences captured may not reflect those less ready to speak. The sample was predominantly female and ethnically homogeneous, limiting transferability to male, non-binary and minoritised survivors whose experiences may differ significantly; future research should explicitly address these underrepresented groups. Interviews took place after the disclosure event, meaning accounts are subject to recall bias; participants’ descriptions reflect retrospective recollections (ranging from 10 months to 9 years) rather than real-time experience, which whilst appropriate to a phenomenological approach, may be shaped by memory reconstruction over time, and complementary ethnographic methods could yield further insight into disclosure as it unfolds. All participants were recruited from a single regional university, meaning they share a relatively similar social and geographic context, which may not reflect the experiences of survivors in other settings with differing access to services and support networks. Finally, this study was conducted within a single national context, which may have shaped participants’ openness to disclosure, experiences of stigma and responses from nurses, and findings may not transfer to cultural contexts with different norms around sexual violence disclosure.

## Implications

### Nurse education

Findings point to a significant and systemic gap in nurse education around SA disclosure. Pre- and post-registration nursing curricula must explicitly equip nurses to communicate belief, recognise and challenge rape myth adherence and understand the profound impact of dismissive or disbelieving responses. Given that disclosure may occur across any clinical setting, mandatory, standardised content on SVA and abuse should be embedded across all pre-registration nursing programmes, with targeted post-registration development for care settings where disclosure is most likely.

### Nurse practice

These findings reframe disclosure as an ongoing, relational process extending before, during and after the moment of telling, rather than a discrete clinical event. In practice, this means nurses must prioritise survivor choice at every stage: communicating clearly about options, explicitly seeking permission before procedures, respecting decisions to decline interventions and remaining attentive to subtle verbal and non-verbal cues that shape whether survivors feel believed and safe.

### Healthcare systems and policy

Organisational policies and procedures must move beyond rhetorical commitments to trauma-informed care and embed survivor-centred approaches structurally, including permission-seeking and informed choice. Current systems, designed around institutional and procedural efficiency, require redesign to restore agency and choice to survivors, reducing the risk of institutional re-traumatisation.

### Further research

Future research should purposively recruit currently underrepresented groups, including male, non-binary, older and minoritised survivors, whose experiences of disclosure may differ substantially and involve compounding barriers. Methodologically, approaches extending beyond retrospective phenomenology, such as longitudinal or ethnographic designs, could capture disclosure as it unfolds in real time. At a broader level, research is needed to evaluate the impact of trauma-informed training interventions on nursing practice and survivor outcomes at scale.

## Conclusion

This phenomenological study is the first to explore the profound complexity of survivors’ experiences disclosing SA to nurses. Characterised by unpreparedness and persistent uncertainty, disclosure represents a vulnerable, emotionally overwhelming experience in which survivors’ fundamental need for belief and validation is paramount. Nurses’ responses profoundly shape disclosure experiences and survivors’ ongoing engagement with healthcare.

The implications extend beyond areas that are high risk for disclosure (sexual health, emergency care, primary care) to all areas of practice where disclosure of sensitive, traumatic experiences may occur. Understanding the phenomenological dimensions of disclosure, the emotions, embodiment, meanings and intersubjective dynamics involved equips nurses to respond with greater sensitivity and effectiveness across clinical contexts.

**Key points for policy, practice and research**

1. Disclosure is a temporally complex, survivor-centred process, not a single moment: Survivors experience disclosure as extending before, during and after the act of telling, characterised by profound unpreparedness and pervasive uncertainty. This reframes how healthcare systems should conceptualise and respond to disclosure, not as a contained clinical event to be processed efficiently, but as an ongoing, emotionally charged experience requiring sustained, relational support. For nursing practice, this means that care cannot end at the point of telling; nurses must be prepared to support survivors across the entire disclosure trajectory.
2. Belief is the cornerstone of survivor-centred nursing care: Fear of not being believed pervaded every stage of participants’ experiences, and nurses’ responses, including subtle non-verbal cues, profoundly shaped whether survivors felt safe to disclose and seek further help. When belief was communicated, survivors could begin to process their experience; when disbelief was perceived, even implicitly, survivors were silenced and deterred from future help-seeking. This has direct implications for pre- and post-registration nurse education, which must explicitly equip nurses to communicate belief, challenge rape myth adherence and understand the devastating consequences of dismissive responses.
3. Trauma-informed care must go beyond rhetoric; agency and choice are non-negotiable: Survivors frequently experienced loss of agency in post-disclosure procedures, mirroring the loss of control central to the assault itself and constituting a form of institutional re-traumatisation. Current healthcare systems are designed around procedural and institutional need rather than survivor need. This has clear policy implications: organisations must redesign disclosure pathways to embed explicit permission-seeking, informed choice and the right to decline procedures at every stage, in line with both trauma-informed care principles and the NMC’s person-centred care standards.
4. Nurse education is failing survivors, and the gap is systemic: Participants’ accounts revealed that nurses lacked the knowledge, skills and confidence to respond effectively to SA disclosure, with training gaps spanning communication, myth awareness and procedural sensitivity. This aligns with wider evidence that HCPs receive little or no formal training in responding to sexual violence. There is a compelling case for mandatory, standardised SVA content across all pre-registration nursing programmes, and for ongoing post-registration development, particularly in mental health, emergency and primary care settings where disclosure is most likely to occur.
5. Future research must address intersectionality and diversity in disclosure experiences: The sample was predominantly White and female, limiting transferability to male, non-binary, older and minoritised survivors, groups facing additional, compounding barriers to disclosure. Future research should purposively recruit these underrepresented populations, and methodologies should be expanded beyond retrospective phenomenology to include longitudinal and ethnographic approaches that capture disclosure in real time. At a broader level, research is needed to evaluate the impact of trauma-informed training interventions on nursing practice and survivor outcomes at scale.

## Supplemental Material

sj-docx-1-jrn-10.1177_17449871261476617 – Supplemental material for Living through disclosure: a descriptive phenomenological exploration of adult survivors’ experiences of disclosing sexual assault to nurses Supplemental material, sj-docx-1-jrn-10.1177_17449871261476617 for Living through disclosure: a descriptive phenomenological exploration of adult survivors’ experiences of disclosing sexual assault to nurses by Claire Dosdale, Mark Bevan and Katy Skarparis in Journal of Research in Nursing
