# Supplementary material for: Living through disclosure: a descriptive phenomenological exploration of adult survivors’ experiences of disclosing sexual assault to nurses
Source: J Res Nurs. 2026 Sep 18:17449871261476617. Online ahead of print. doi: 10.1177/17449871261476617 (PMC13588952; doi:10.1177/17449871261476617)
Supplement: Supplementary file 1 [file sj-docx-1-jrn-10.1177_17449871261476617.docx]

# COREQ Checklist

Consolidated Criteria for Reporting Qualitative Research (Tong, Sainsbury & Craig, 2007)

**Manuscript: Living Through Disclosure: A Phenomenological Exploration of Adult Survivors' Experiences of Disclosing Sexual Assault to Nurses**

| **No.** | **Topic** | **Guiding Question** | **Notes** |
| --- | --- | --- | --- |
| **Domain 1: Research Team and Reflexivity** | | | |
| **Personal Characteristics (Items 1–5)** | | | |
| 1 | **Interviewer/facilitator** | Which author/s conducted the interview or focus group? | XXXXXX |
| 2 | **Credentials** | What were the researcher's credentials, e.g. PhD, MD? | XXXXXXX– BSc, MSc  XXXXXX) – PhD  XXXXXX) - PhD |
| 3 | **Occupation** | What was their occupation at the time of the study? | Assistant Professor (XX,XX,XX) |
| 4 | **Gender** | Was the researcher male or female? | Female |
| 5 | **Experience and training** | What experience or training did the researcher have? | XXXXX - Previous research experience undertaking MSc, Methodological CPD training. This is PhD study. |
| **Relationship with Participants (Items 6–8)** | | | |
| 6 | **Relationship established** | Was a relationship established prior to study commencement? | NO |
| 7 | **Participant knowledge of the interviewer** | What did participants know about the researcher, e.g. personal goals, reasons for doing the research? | Rational for research was explained. Participants were told researchers clinical background (sexual health specialist nurse) to demonstrate insight into interest in topic. |
| 8 | **Interviewer characteristics** | What characteristics were reported about the interviewer/facilitator, e.g. bias, assumptions, reasons and interests in the research topic? | Researcher had an interest in understanding experiences of disclosure in healthcare setting. |
| **Domain 2: Study Design** | | | |
| **Theoretical Framework (Item 9)** | | | |
| 9 | **Methodological orientation and theory** | What methodological orientation was stated to underpin the study, e.g. grounded theory, discourse analysis, ethnography, phenomenology? | Methods: Husserlian descriptive phenomenology; Giorgi (2012); XXXXX (2014) stated explicitly |
| **Participant Selection (Items 10–13)** | | | |
| 10 | **Sampling** | How were participants selected, e.g. purposive, convenience, consecutive, snowball? | Methods: purposive sampling stated |
| 11 | **Method of approach** | How were participants approached, e.g. face-to-face, telephone, mail, email? | Methods: recruited via regional HEI via email advert |
| 12 | **Sample size** | How many participants were in the study? | Methods: n=9 survivor participants stated |
| 13 | **Non-participation** | How many people refused to participate or dropped out? Reasons? | Nil. Those who refused to participate did not respond to the advert. |
| **Setting (Items 14–16)** | | | |
| 14 | **Setting of data collection** | Where was the data collected, e.g. home, clinic, workplace? | All interviews took place on university campus in privately booked rooms. |
| 15 | **Presence of non-participants** | Was anyone else present besides the participants and researchers? | No. |
| 16 | **Description of sample** | What are the important characteristics of the sample, e.g. demographic data, date? | age range (19–51), gender (8F, 1M) stated; ethnicity limited — acknowledged in Limitations |
| **Data Collection (Items 17–23)** | | | |
| 17 | **Interview guide** | Were questions, prompts, guides provided by the authors? Was it pilot tested? | Open questions with natural expansion on conversation led by participants. No pilot testing. |
| 18 | **Repeat interviews** | Were repeat interviews carried out? If yes, how many? | Nil. |
| 19 | **Audio/visual recording** | Did the research use audio or visual recording? | audio-recorded |
| 20 | **Field notes** | Were field notes made during and/or after the interview or focus group? | reflexive journal post interview |
| 21 | **Duration** | What was the duration of the interviews or focus group? | 60–150 minutes |
| 22 | **Data saturation** | Was data saturation discussed? | phenomenological sufficiency/data saturation not applicable in descriptive phenomenology. |
| 23 | **Transcripts returned** | Were transcripts returned to participants for comment and/or correction? | member checking not used; rationale provided (re-traumatisation risk, consistent with Giorgi) |
| **Domain 3: Analysis and Findings** | | | |
| **Analysis (Items 24–28)** | | | |
| 24 | **Number of coders** | How many data coders coded the data? | 1 |
| 25 | **Description of coding tree** | Did authors provide a description of the coding tree? | Figure 1: thematic structure provided; 68 essential structures, 2 themes, 13 subthemes reported |
| 26 | **Derivation of themes** | Were themes identified in advance or derived from the data? | Derived from data |
| 27 | **Software** | What software, if applicable, was used to manage the data? | Nil used |
| 28 | **Participant checking** | Did participants provide feedback on the findings? | member checking not used; rationale clearly stated |
| **Reporting (Items 29–32)** | | | |
| 29 | **Quotations presented** | Were participant quotations presented to illustrate the themes/findings? | Yes — Findings: extensive participant quotations presented throughout with pseudonyms |
| 30 | **Data and findings consistent** | Was there consistency between the data presented and the findings? | Yes — Findings: quotations clearly mapped to subthemes and themes throughout |
| 31 | **Clarity of major themes** | Were major themes clearly presented in the findings? | Yes — Findings: two themes, 13 subthemes clearly structured; Figure 1 provides visual overview |
| 32 | **Clarity of minor themes** | Is there a description of diverse cases or discussion of minor themes? | Findings: intersectional diversity (gender, age, ethnicity) noted in Limitations; limited minor theme discussion in findings body |

Reference: Tong A, Sainsbury P, Craig J (2007) Consolidated criteria for reporting qualitative research (COREQ): a 32-item checklist for interviews and focus groups. International Journal for Quality in Health Care 19(6): 349-357.
